# Heat Health Messages: A Randomized Controlled Trial of a Preventative Messages Tool in the Older Population of South Australia

**DOI:** 10.3390/ijerph14090992

**Published:** 2017-08-31

**Authors:** Monika Nitschke, Antoinette Krackowizer, Alana L. Hansen, Peng Bi, Graeme R. Tucker

**Affiliations:** 1 Department for Health and Ageing, 11 Hindmarsh Square, Adelaide, SA 5000, Australia; grtucker@adam.com.au; 2 School of Public Health, The University of Adelaide, Adelaide, SA 5005, Australia; antoinette.krackowizer@adelaide.edu.au (A.K.); alana.hansen@adelaide.edu.au (A.L.H.); peng.bi@adelaide.edu.au (P.B.)

**Keywords:** heat-related illness, randomized trial, older people, prevention

## Abstract

This study explores the efficacy of providing targeted information to older individuals to prevent adverse health outcomes during extreme heat. Participants ≥65 years of age (*n* = 637) were recruited from previous population-based studies and randomized into intervention and control groups. The intervention group received evidence-based information leaflets and summarised “Beat the Heat” tips. Post summer 2013–2014, participants responded to questions about their behaviours and their health experiences. Chi square analysis and risk ratios (RR) were used to determine the difference in effects. Responses were received from 216 intervention subjects and 218 controls. Behaviour modification during extreme heat was similar in both groups except for significant increases in the use of cooling systems and the use of a wet cloth to cool the skin in the intervention group. Both actions were recommended in the information package. More people in the intervention group also claimed to have had adequate heat health information. After adjusting for confounders, the RR for self-reported heat stress experienced during summer 2014 indicated a 63% (RR 0.37; 95% CI: 0.22–0.63) reduction in the intervention group compared to the control group. Access to intensive prevention information may have contributed to this positive outcome, indicating the potential usefulness of targeted heat-health information for seniors.

## 1. Introduction

Health problems associated with extreme hot weather over several days should be preventable, but recent findings indicate that vulnerable people are at risk during heat, especially those with co-morbidities, and older people [1]. Furthermore, it has been shown that there is a high risk of a poor long term health outcome for older people once they have succumbed to heat stroke [2]. Recent research in Adelaide, South Australia, found that older people have a higher risk of renal and direct heat related health problems during times of extreme heat than younger age groups [3,4]. 

Following extreme heat waves in major cities around the world, heat wave warning systems have been implemented with the aim to reduce mortality and morbidity [5,6,7]. While evaluations of these interventions are still sparse, some studies provide evidence that early warnings may be effective in saving lives and reducing morbidity [5,7,8].

The messages that are used during extreme heat warnings are based on common understanding of the human physiology and the specific risk factors that can impact during extreme heat. For example, evaporation (sweating) is a prerequisite of cooling down the core body temperature. As we grow older, this function is reduced due to ageing, chronic diseases and the medication associated with these diseases [1]. Other risk factors may arise when older people live alone, have impaired mobility, and do not use air conditioners due to economic concerns, during extreme and prolonged heat [9,10]. A case-control study specific for Adelaide revealed that living alone and having pre-existing heart disease was significantly associated with mortality, whilst having air conditioning in bedrooms offered significant protection [11]. 

Recent summers in Australia have been extremely hot, with extreme temperatures extending over many days. The Adelaide summer of 2009 was record breaking. During the 13 day heatwave, there were five subsequent days with maximum temperatures above 41 °C and no substantial cooling overnight [3]. Following the 2009 heat wave, extreme heat warnings were initiated due to the fact that unprecedented excess morbidity and mortality had been experienced during this event. The threshold for extreme heat warnings in Adelaide is a three-day rolling forecast of average daily (minimum and maximum) temperatures of 32 °C or above (for example 40 °C daytime and 24 °C night-time). During the 2014 summer, extreme heat warnings were issued twice [7,12].

A previous population-based survey in the older population in South Australia indicated that a high percentage of respondents were using some adaptive behavior during hot days, but when directly asked about their actions following extreme heat warnings, a sizable group (53.5%) answered that they did not change their behavior [9]. While this survey did not explore the reasons for this, a qualitative study in London suggested that older people may not consider themselves to be old or vulnerable during extreme heat [13]. Pre-existing chronic disease, need of household help and reduced mobility predicted health outcomes during hot periods in this population. The findings indicated that older people may benefit from more intense interventions in the form of targeted education [9]. 

To date, global research shows a good understanding of the problems concerning older people’s risk factors, behaviours and perceptions during extreme heat, but is still lacking evidence of how to assist successful adaptation. The aim of the Heat Health Messages study was to trial the efficacy of heat-health messages sent out at the beginning of summer and their health impact. The study’s objective was to test an intervention tool that is based on known risk factors in South Australia and heat-health information relevant to older people in a randomized trial. If successful, this tool could be used in the future to reduce adverse health outcomes in older people during hot summers.

## 2. Materials and Methods 

### 2.1. Collaboration

The Heat Health Messages study was guided by a multidisciplinary team of key leaders from across relevant government and non-government agencies working in the field of extreme heat and health prevention and with experience in developing adaptive responses for individual well-being, community health and the emergency sector. They provided their knowledge, practical understanding and valuable advice to the project. 

Ethical approval was granted from the University of Adelaide’s Ethics Committee and SA Health’s Human Research Ethics Committee (HREC/13/SAH/110).

### 2.2. Eligible Population and Recruitment

**Figure 1:**
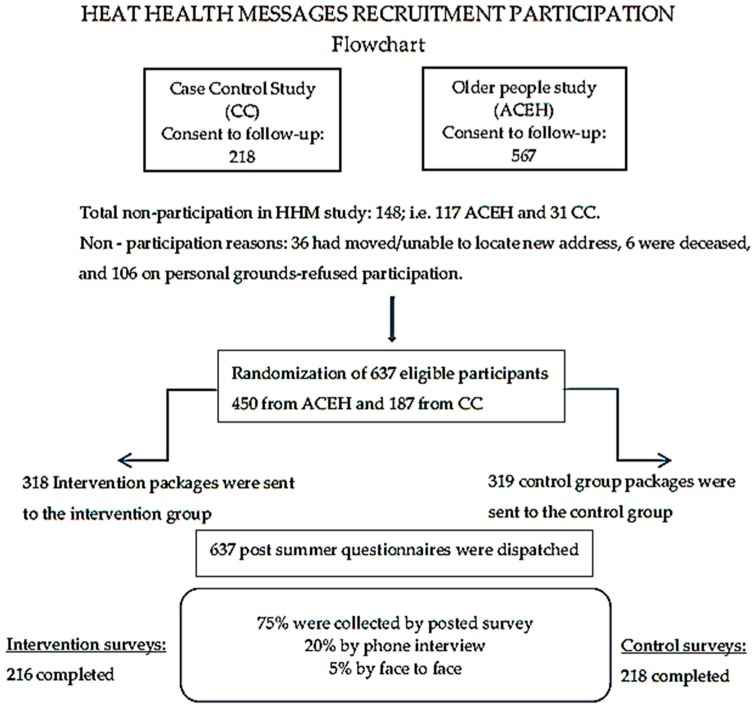
Flowchart of recruitment, randomization and study processes.

The processes involved selection of the eligible population, recruitment, randomization of participants into either intervention or control groups, as well as distribution and collection of questionnaires as documented in the flow chart (Figure 1).

The eligible population was drawn from two previously completed studies. Firstly, from a Case Control study (CC) [11,14] in which risk factors for heat susceptibility in control subjects from the community were statistically compared to those of patients who were admitted to hospital, or died during the 2009 extreme heat wave in Adelaide. From the CC control group, people aged ≥65 years of age were invited (218 subjects) to participate in the present study. Secondly, from a study which investigated the adaptive capabilities of the elderly (≥65 years of age) during recent extreme heat events (ACEH) in SA (567 subjects) [9]. The participants of both studies had previously been selected randomly from large data bases: CC from the electoral roll (metropolitan only) and ACEH from the electronic phone book of SA. Both populations had previously consented (during their respective phone interviews) to take part in future studies on heat and health. This prior process was advantageous in that participants had already established a trusted relationship with the researchers. Thus, the eligible population for the present study was gathered without the need for an extra recruitment strategy and provided an ideal population upon which to base the investigation. As the study populations in these studies were representative of the state’s older population, the findings of the present study are also generalizable to the population of older people in SA. 

The recruitment process commenced with an initial phone contact to all potential participants (Figure 1), briefly explaining the HHM study and confirming whether consent to participate in a further study remained valid. Of the 785 eligible people, 106 declined, 6 had died and 36 could not be contacted. In total, 637 consenting participants were informed that the study was soon to commence and a letter of invitation containing further details was to follow. The cohort was then randomized into the intervention (318) and control groups (319). The participants were entered into a data base and electronically randomized into the two groups (intervention or control) using the Stata 13 statistical package “set seed” command [15].

### 2.3. Intervention Materials and Questionnaire 

The trial began on the 1 November 2013 when all study information had been sent out to the intervention and control groups. It ended on the 15 March 2014 after participants had received the end of study survey questions. Participants in the control group were sent an introductory letter advising them to take notice of: public announcements, warnings and messages about extreme heat, fires, electricity supply outages or blackouts over the coming summer, and to seek medical advice when feeling unwell or if taking medications. 

Participants in the intervention group were sent an introductory letter accompanied by the intervention package containing extra information. The intervention package comprised an information sheet that provided information on how to deal with extreme heat conditions; a “Top Tips Heat-Health Card” (this laminated card was to accompany the “Beat the Heat” Fridge Magnet) (to be viewed in the Supplementary Materials); the SA Health Department’s “Extreme Heat Booklet—a guide to coping and staying healthy in the heat”; three SA Health advice fact-sheets (“Advice For An Older Person”, “Caring For An Older Person” and ”Safe Food Handling During Extreme Heat”) [16]. 

The end of trial questionnaire included questions assessing respondents’ heat-health behaviours and self-rated health outcomes associated with extreme heat events during the 2013/2014 summer (Supplementary Materials). The questionnaire also covered demographics, environmental setting, health status and medications, availability and use of cooling, heat health knowledge and awareness of heat-related warnings. Completed interviews were received via reply-paid envelope (75%) or by interviews that were conducted either face to face (20%) or over the phone (5%). 

### 2.4. Statistical Methods and Outcome Measures

Prevalence data for demographic results, confounders, behaviour changes during extreme heat, heat-related health outcomes and sources of information for intervention and control groups were calculated and differences between the intervention and control groups assessed with the chi square test in Stata [15].

For comparing study outcomes in the intervention with the control group, risk ratios (RR) were calculated given their preference over odds ratios in randomized trials [17]. Adjusted RR calculation was undertaken using the log binomial regression command [glm y x z, link(log) eform binomial] in Stata [15]. The RR was adjusted by the confounders that were not equally distributed (medication for mental health, health status, aids for walking) between the intervention and the control group. For calculation of the sample size before the trial, a prevalence of 10% in the control group and 20% in the intervention group was assumed. Using two-sided 95% confidence levels and a statistical power of 80%, a total sample size of 438 was required [18].

## 3. Results

As shown in the flowchart (Figure 1) of the initial randomised subjects (318 from the intervention group and 319 controls), 216 and 218, respectively, completed the questionnaires by the end of summer 2013/2014. The participation rate post randomization was 68% and similar for the two groups. Thirty-three of the questionnaires were returned to sender unopened.

Table 1 indicates that the demographics were similar for the two groups. The mean overall age was 79.9 years (data not shown) with a minimum age of 65 and a maximum of 102. The average age of the intervention group was 79.4 and 80.3 in the control group. Overall, the demographic distribution was similar in both groups.

Table 2 compares the distribution of possible confounding variables which assess participants’ pre-existing disease status. Of these, health status, use of an aid for walking and taking medication for mental health were not equally distributed (borderline significance = *p* < 0.10) between the two groups; all three variables were of higher proportion in the intervention group. 

Table 3 shows the numbers and percentages of participants undertaking behavioral changes during extreme heat or hot weather. The data shows that for most of the potential modifying behaviors during heat, there was little difference between the two groups. However, three statistically different behavior modifications were observed. Air conditioner (A/c) use (most times to always) during hot weather was significantly higher in the intervention group (74.4%) compared to the control group (63.4%). This increase was despite the fact that there was similar prevalence of home a/c in each group (96% vs. 97%). The percentage of participants who were concerned about the cost of running an A/c was also similar, albeit slightly higher in the intervention group (44% vs. 39%). More participants in the intervention group (16% vs. 8%) used a wet cloth on their face, neck or body (most of the time to always) to cool down during heat waves. Finally, more people in the intervention group believed that they had enough information to “beat the heat” (94% vs. 88%). 

Table 4 displays the distribution of health outcomes in each group during “the heat” of the summer 2013/2014. A higher prevalence of dizziness (20% vs. 13%; *p* < 0.1) and headaches (28% vs. 20%; *p* < 0.5) was observed in the intervention group. Heat stress, on the other hand, was significantly reduced in the intervention group (8% vs. 19%; *p* < 0.01) compared to the control group. 

Health outcomes were adjusted for the confounding variables (mental health-related medication, health status and use of aids for walking, listed in Table 2). Table 5 shows the crude or unadjusted RR and the adjusted RR for health outcomes experienced during extreme heat. The table indicates that, in the univariate analysis, the risk ratio for headache was increased in the intervention group and there was a significant protective effect for heat stress. After adjustment by the confounders, only heat stress remained significant.

## 4. Discussion

This is the first randomized controlled trial evaluating the effect of an information package on behavior change and health outcomes related to extreme heat in a population of older people in Australia. To the best of our knowledge, we maintain that, worldwide, there is only one other study that has trialed the effectiveness of intervention by targeted information on extreme heat. In Japan, a community trial allocated warnings via community-specific information channels (*n* = 397) or, in another group, the warnings plus supplying water bottles (*n* = 284) with useful messages; the control group (*n* = 391) receiving neither [19]. Similar to our study, the Japanese trial aimed to find out whether heat wave warnings were heeded in a population-based sample of older people aged 65 to 84 years of age, thus leading to behavior changes in the intervention groups. Both studies successfully showed behavior changes; our study also provided evidence of reduced heat-related health outcomes in the intervention group compared to the control group.

The older people enrolled in our study had been previously randomly selected from large population data sets, and therefore the results from this study are generalizable to the general population of people 65 and older in SA. Unlike the control group, the intervention group received material that provided in depth information on the potential health risks to older people during heat and recommended ways to avoid falling ill.

The main findings in our study indicated that participants in both groups generally made very similar decisions about their behavior during extreme heat. A high percentage engaged in preventive actions that assisted the reduction of body temperature. This is not surprising because the control group was told in the study information letter to listen to media announcements about extreme heat warnings and other emergency messages and this may have assisted in their acquisition of heat health knowledge. However, there were two behaviors that were significantly higher in the intervention group, i.e., “use of a cooling system” (always–most of the time vs. sometimes–never) and placing a wet cloth on face, neck or body’ (always–most of the time vs. sometimes–never). Both messages were specifically included on the laminated “top tips to beat the heat” card (with a magnet suitable for attaching to a refrigerator door) as part of the intervention package (view in Supplementary Materials). The suggestion to use a wet cloth is not included in the extreme heat warnings messages for the public, and was therefore not available to the control group. The top tips also emphasized using A/c during extreme heat periods. The cost of running an A/c was clearly of concern in both groups, however slightly more so, in the intervention group. Nevertheless, the intervention group had a significantly higher prevalence of using their cooling system compared to the control group.

Health outcomes during extreme heat were central to the assessment of the success of this intervention. Adjustment by unequally distributed confounders ensured that outcomes were truly associated with the intervention. While the proportions for “dizziness” (*p* < 0.1) and “headaches” (*p* < 0.05) were increased in the intervention group, adjusted RRs were not significantly increased.

The only health outcome which was significantly reduced and therefore indicative of a positive result of the intervention was heat stress. Based on the adjusted RR, the reduction in the intervention group was 78%, a sizable reduction for a health outcome which is on the trajectory to more serious heat-related illnesses such as dehydration and heat stroke. During the 2009 heatwave in Adelaide, direct heat-related (ICD-10 E86, T67, X30) hospital and emergency admissions were significantly increased, with more than half of the 518 excess cases being 65 years of age and older [3]. In a case-control study, we later interviewed patients with heat-related admissions during the 2009 heatwave [11,14]. Findings showed that pre-existing dementia and heart disease had significantly increased their risk of hospitalization. Heat stroke is associated with poor recovery and is more likely to occur in older people, due mainly to co-morbidities and medications, but also because they are less likely to feel the heat, have reduced sweat output and a decreased sense of thirst [2]. Reminding older people of their vulnerabilities and giving them advice is therefore a useful protective measure as well as a cost-effective intervention. 

In the present study, the two behavioral changes found to be increased in the intervention group may have assisted in the reduction of falling ill to heat stress. A/c use and applying a wet cloth are two measures that can assist in cooling down when temperatures are in excess of 35 °C, particularly so for older people whose thermoregulation may be compromised for various reasons. An international review of risk factors of health during heat waves confirmed that cooling measures such as those suggested in this study were instrumental in lowering the risk of death [20]. 

There were also more participants in the intervention group who believed that they had enough information on how to deal with extreme heat, thus suggesting that the intervention package guided the subjects towards a more cautionary approach to extreme heat by embracing the advice given. 

There are some limitations to this study. Randomization, in theory, should result in groups that are equivalent, but differences can emerge, especially if the sample size of the trial is small. In this study, differences between the groups were observed. While demographic and environmental variables were equally distributed, higher (of borderline significance) prevalence of pre-existing ill-health was detected in the intervention group for health status, aids for walking and taking mental health-related medication; this was overcome in the analysis by adjusting for these unequally distributed confounders. One of the reasons for this to occur could have been the loss of 30% of initial responders; this attrition has the potential to contribute to attrition bias. Furthermore, some bias may have been introduced through self-selection into this study from an originally randomly selected population of people aged 65 and over which may have affected the generalizability of the results of this study. In this study, health outcome responses were by self-assessment leading to less valid outcomes than if a doctor had undertaken a clinical examination. This can only be remedied in the future by conducting intervention trials in a clinical setting. 

Studies such as these provide evidence about relevant interventions aimed to lower the health risks of climate change in the community [21]. Further trials could shed light on other useful adaptation measures to increased heat. Examples could include building improvements that enhance thermal comfort, relevant support services for older people and general practitioners’ involvement in assessing older individuals’ ability to remain healthy during extreme heat. 

## 5. Conclusions

This randomized controlled intervention study of behaviors and health effects during the 2013/2014 summer contributes further to the overall risk assessment of heat waves in SA. The findings show a clear reduction in heat stress, one of the direct indicators of heat-related illness that can prove fatal if left unchecked. It is likely that advice to lower the temperature of the home environment with A/c and by personal cooling with the wet cloth technique may have significantly contributed to this success. This type of evidence-based information has the potential to enable older people to stay healthy during periods of extreme heat. As the population ages, a more resilient and well-prepared older population will reduce costs to health and emergency services during extreme heat events, while supporting independent living of older people in South Australia.

## Figures and Tables

**Table 1:** Frequency distribution of demographic variables by intervention and control groups.

Demographic Factors	Intervention *n* = 216 (%)	Controls *n* = 218 (%)
Age group ≥75	137 (63.4)	145 (66.5)
Age group <75	79 (36.6)	73 (33.5)
Females	108 (50.0)	126 (57.8)
Living in a house	158 (73.2)	147 (67.4)
Living in a unit	41 (19.0)	52 (23.9)
Living in a duplex	17 (7.9)	15 (6.9)
Own your accommodation	174 (80.6)	171 (80.7)
Having outdoor blinds, shutters, awnings	124 (57.4)	129 (59.2)
A/c presence	207 (95.8)	212 (97.3)

**Table 2:** Assessment of the frequency distribution of possible confounding variables indicating pre-existing chronic disease by intervention and control groups.

Factors	Intervention *n* = 216 (%)	Controls *n* = 218 (%)
Health status		
Good to excellent (compared to fair-poor)	152 # (70.4)	168 # (77.4)
Aid for walking	75 # (34.7)	59 # (27.1)
Medication for		
Diabetes	28 (13)	33 (15.1)
Thyroid	24 (11.1)	27 (12.4)
High blood pressure	127 (58.8)	138 (63.3)
Heart failure	12 (5.6)	8 (3.7)
Other heart problems	66 (31)	53 (24.3)
Renal	8 (3.7)	6 (2.8)
Respiratory	30 (14)	35 (16.1)
Mental health	43 # (20)	28 # (28)
No medication	28 (13)	29 (13.3)
Don’t know	2	3

# *p* < 0.1 = borderline; * *p* < 0.05; ** *p* < 0.001.

**Table 3:** Assessment of the frequency distribution of behavior during extreme heat by intervention and control groups.

Factors	Intervention Count 216 (%)	Controls Count 218 (%)
Cooling the house		
Use of outside shades	122 (98.4)	127 (98.5)
Use of inside shades	204 (94.4)	196 (89.9)
Use of A/c (most times-always)	154 * (71.3)	135 * (61.9)
Cost of A/c is a problem	93 (43.1)	86 (39.5)
Cooling behavior		
Cooling down via shower, bath, swim	46 (21.3)	36 (16.5)
Wearing lighter clothes	178 (82.4)	177 (81.2)
Using wet cloth (most-always)	34 * (15.7)	18 * (8.3)
Stayed indoors	189 (87.5)	192 (88.1)
Let cool breeze in	177 (81.9)	182 (83.5)
Drinking lots more fluids	87 (40.3)	99 (45.4)
Concerned about pets	66 (78.6)	68 (81.9)
Had enough “heat” information	201 * (94.4)	188 * (88.3)
Concerns during heat	121 (56.0)	116 (53.2)
Did things different this summer	67 (31.0)	82 (37.6)
Had or made contact during hot weather	128 (59.3)	124 (56.9)
Was well prepared for extreme heat	208 (96.3)	207 (95.0)

# *p* < 0.1 = borderline; * *p* < 0.05; ** *p* < 0.001.

**Table 4:** Assessment of the frequency distribution of health outcomes during extreme heat by intervention and control groups.

Factors	Counts *n* = 434	
	Intervention *n* = 216 (%)	Controls *n* = 218 (%)
Heat health aspects		
Needed help during heat	9 (4.2)	9 (4.1)
Needed help from a doctor during heat	5 (2.5)	11 (5.1)
Affected by hot weather	61 (28.4)	62 (29.1)
Experience during hot weather		
Anxiety	22 (10.2)	22 (10.1)
Loss of balance/dizzy	43 # (19.9)	29 # (13.3)
Fall	5 (2.3)	9 (4.2)
Headache	61 * (28.2)	43 * (19.7)
Shortness of breath	38 (17.6)	35 (16.1)
Heat stress	17 * (7.9)	41 * (18.8)
Heart condition	11 (5.1)	6 (2.8)
Renal problems	2 (0.9)	4 (1.8)
Something else	44 (20.5)	36 (16.5)

# *p* < 0.1 = borderline; * *p* < 0.05; ** *p* < 0.001.

**Table 5:** Crude and adjusted risk ratios and 95% Confidence Intervals (95% CI) expressing the health outcomes in the intervention compared to the control group. For adjustment, the following confounders from Table 2 were included: mental health-related medication, health status and use of aids for walking.

Health Outcomes	Crude Risk Ratio (95% CI)	Adjusted Risk Ratio (95% CI)
Anxiety	1.01 (0.54–1.75)	0.76 (0.45–1.28)
Dizziness	1.62 (0.97–2.71) #	1.24 (0.81–1.90)
Falls	1.85 (0.61–5.62)	1.48 (0.51–4.30)
Headache	1.60 (1.03–2.50) *	1.26 (0.91–1.73)
Respiratory	1.12 (0.67–1.85)	0.92 (0.61–1.41)
Heat stress	0.37 (0.20–0.67) *	0.37 (0.22–0.63) **
Heart	0.54 (0.2–1.49)	0.43 (0.17–1.13) #
Renal	0.50 (0.09–2.77)	0.42 (0.08–2.20)

# *p* < 0.1 = borderline; * *p* < 0.05; ** *p* < 0.001.

## Supplementary Materials

The following are available online at www.mdpi.com/1660-4601/14/9/992/s1. Intervention material and end of season questionnaire.
