# Supplementary material for: Heat Health Messages: A Randomized Controlled Trial of a Preventative Messages Tool in the Older Population of South Australia
Source: Int J Environ Res Public Health. 2017 Aug 31;14(9):992. doi: 10.3390/ijerph14090992 (PMC5615529; doi:10.3390/ijerph14090992)
Supplement: Supplementary file 1 [file ijerph-14-00992-s001.pdf]

International Journal of
Environmental Research and Public Health

MDPI

Supplementary Materials

Heat Health Messages: A Randomized Controlled Trial of a Preventative Messages Tool in the Older Population of South Australia

Monika Nitschke, Antoinette Krackowizer, Alana Hansen, Peng Bi and Graeme Tucker

1. Beat the Heat Top Tips and Fridge Magnet

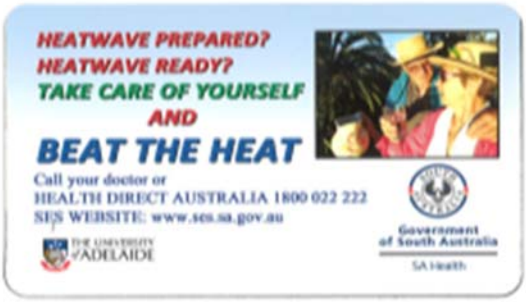

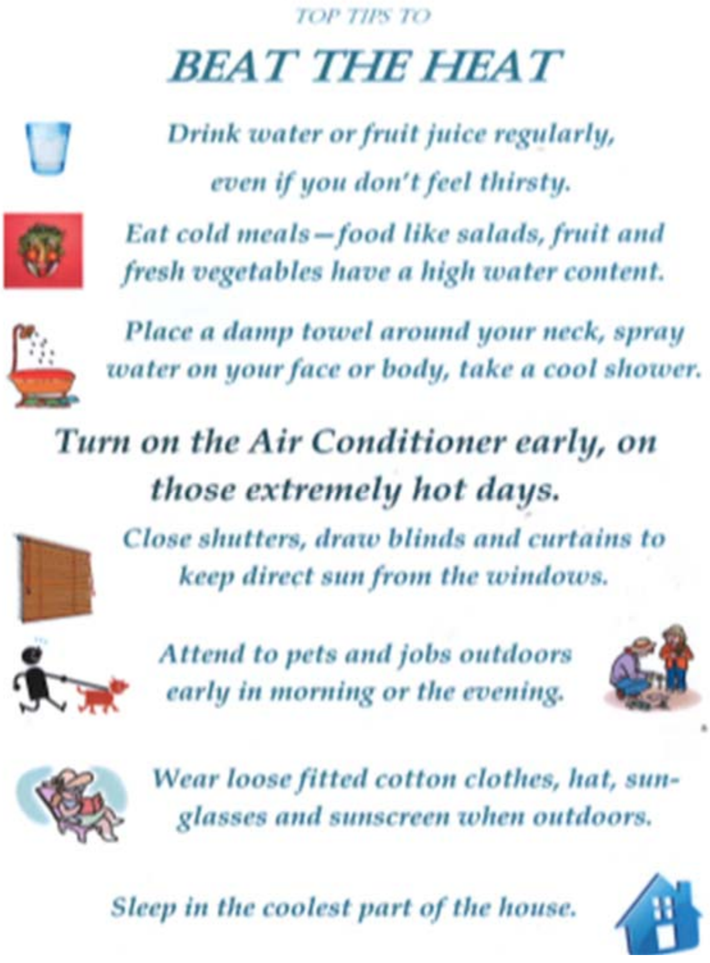

TOP TIPS TO

BEAT THE HEAT

Symptoms of heat stress include:

- ⇒ Having a headache
- ⇒ Feeling dizzy
- ⇒ Feeling weak or faint
- ⇒ Feeling of nausea
- ⇒ Vomiting
- ⇒ Having muscle cramps

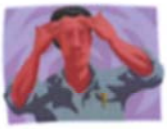

If you feel ill, do not hesitate to

SEEK MEDICAL ADVICE IMMEDIATELY:

Call family/a friend /a neighbour
PH________________________________

Call your doctor PH________________________

Call Health Direct Australia PH 1800 022 222

Call an AMBULANCE PH 000

More website information from:

SES: www.ses.sa.gov.au

SA Health: www.sahealth.sa.gov.au

THE UNIVERSITY of ADELAIDE

Government of South Australia
SA Health

2. End of summer season questionnaire

Government of South Australia
SA Health

HHM 2014

THE UNIVERSITY OF ADELAIDE AUSTRALIA

HEAT HEALTH MESSAGES RESEARCH 2014

INTRODUCTION

Thank you for participating in the research conducted by the University of Adelaide.

The survey will assess how people 65 years or older, coped during hot weather this summer. We will ask general questions as well as questions about your home and your health.

Your participation will assist us in learning more about problems encountered and how you dealt with those problems during the extreme heat of summer 2013/14.

We will remove all information that could identify you and then collate the answers from all participants that will then be presented in a report. We assure you that all information you give us will remain confidential.

The questionnaire will take approximately 15 minutes to complete, but take as much time as you need to answer the questions that are especially relevant to you.

Once you have completed the questions, please return them in the reply paid envelope provided, to Antoinette the Research Project Officer:

SA HEALTH
Ms Antoinette Krackowizer, ENVIRONMENT
REPLY PAID
PO BOX 6
ADELAIDE SA 5000

If there are problems in answering the study questions, do not hesitate to contact her:
Antoinette at SA Health, Ph. 8226 7143
antoinette.krackowizer@health.sa.gov.au

Your participation is voluntary and you can choose not to answer any particular question or any section. You are also free to withdraw from the study at any time. If you have further questions, wish to raise concerns or discuss any aspect of the study; please contact either the Associate Researcher:

Dr Monika Nitschke, Ph 8226 7126
monika.nitschke@health.sa.gov.au

Alternatively, the Chief Investigator:
Prof Peng Bi,
Discipline of Public Health,
University of Adelaide;
Ph. 8313 3583 peng.bi@adelaide.edu.au,
To discuss matters related to the University policy on research involving human participants or your rights as a participant, contact,

The Human Research Ethics Committee's Secretary, Ph. 8313 6028 or visit:
http://www.adelaide.edu.au/ethics/human/guidelines/applications/#complaint.

Thank you for your help.

Sincerely

Professor Peng Bi MBBS PhD
Discipline of Public Health
University of Adelaide

3. Questionnaire

Government of South Australia
SA Health

THE UNIVERSITY OF ADELAIDE AUSTRALIA

HHM 2014

PLEASE TICK THE APPROPRIATE BOX, OR WHERE REQUIRED, WRITE YOUR ANSWER.

A. Introduction

A.1 Do you live independently in a....

- ☐ House
- ☐ Unit, flat, apartment
- ☐ Duplex, semi-detached house
- ☐ Other (explain)...........................

A. 2 Do you ...

- ☐ Own your home or
- ☐ Rent

A. 3 If you rent, is it ...

- ☐ Government Rental or
- ☐ Private Rental

B. Health Status

B.1 In general, would you say that your health is? (Read the Options and tick one answer)

- ☐ Excellent
- ☐ Very good
- ☐ Good
- ☐ Fair
- ☐ Poor
- ☐ Not sure

B.2 Do you regularly take doctors' prescribed medications for any of the following conditions?
(Read the Options and tick the ones you have)

- ☐ Diabetes
- ☐ Thyroid disease
- ☐ High blood pressure
- ☐ Heart failure
- ☐ Other heart problem (e.g. heart attack, stroke, angina)
- ☐ Kidney or Renal problem
- ☐ Respiratory problem (E.g. asthma, COPD)
- ☐ Depression, anxiety, memory loss or other mental health illnesses
- ☐ Don't know
- ☐ No, I don't take any tablets or medications prescribed by a doctor

B.3 Do you use any of the following aides for getting around?
(Read the Options and tick all the ones that apply to you)

- ☐ Walking aids (frames or sticks, etc.)
- ☐ Wheelchair
- ☐ Gopher / scooter
- ☐ No aides
- ☐ Other (explain)..............................

C. Your House in Summer

These questions are about using air conditioners and other ways of keeping your home cool when the weather is hot.

C.1 Do you have outdoor blinds, shutters or awnings on your windows?
(Tick one answer)

- ☐ Yes
- ☐ No

C.2 Did you use the outdoor blinds, shutters or awnings to shade your windows throughout the hot days this summer?
(Tick one answer)

- ☐ Yes
- ☐ No
- ☐ Not applicable

C.3 During the hot days, did you use your indoor blinds and draw the curtains to block out the sun?
(Tick one answer)

- ☐ Yes
- ☐ No
- ☐ Not applicable

C.4 Do you have any form air-conditioning at home?
(Tick one answer)

- ☐ Yes
- ☐ No
- ☐ Not sure

2

HHM Questionnaire March 2014 HHM documents

C.5 When it was hot this summer, did you use the cooling system?

(Tick one answer)

- ☐ Always
- ☐ Most of the time
- ☐ Sometimes
- ☐ Never

C.6 At which times of the day did you most use the cooling system?

	ON		OFF
Morning	☐	Morning	☐
After lunch	☐	After lunch	☐
Late afternoon	☐	Late afternoon	☐
Evening	☐	Evening	☐
Night time	☐	Night time	☐

C.7 Does the cost of running air conditioning in your home in summer, stop you from using it as much as you would like?

- ☐ No
- ☐ Yes
- ☐ Comment (optional)

....

....

....

....

D. Heatwaves

These next questions are about heatwaves; this is when the temperature is very hot for several days and nights in a row.

D.1 When it was very hot this summer, how often did you cool off by having a cool shower, bath or a swim?

(Tick one answer)

- ☐ Often
- ☐ Most of the time
- ☐ Sometimes
- ☐ Never

D.2 When it is was very hot this summer, how often did you wear lighter, cooler cotton clothing?

(Tick one answer)

- ☐ Always
- ☐ Most of the time
- ☐ Sometimes
- ☐ Never

D.3 To cool off during heat waves this summer, how often did you place a wet cloth on your face, neck or body?

(Tick one answer)

- ☐ Always
- ☐ Most of the time
- ☐ Sometimes
- ☐ Never

D.4 To avoid the sun during heat waves this summer, how much did you stay indoors?

(Tick one answer)

- ☐ Always
- ☐ Most of the time
- ☐ Sometimes
- ☐ Never

D.5 When there was a cooling breeze in the evening, how often did you open up your windows and doors to let in the breeze cool your home?

(Tick one answer)

- ☐ Always
- ☐ Most of the time
- ☐ Sometimes
- ☐ Never

D.6 Did you drink more fluids when the weather was very hot?

(Tick one answer)

- ☐ No
- ☐ Yes, a bit more
- ☐ Yes, lots more

D.7 If you have pets or animals, how concerned are you about their health and welfare during the heat?

(Tick one answer)

- ☐ No concern
- ☐ Little concern
- ☐ Moderate concern
- ☐ Major concern
- ☐ Don't have pets/animals

D.8 Do you think that you had adequate information this summer to cope with the hot days and BEAT THE HEAT?

(Tick one answer)

- ☐ No
- ☐ Yes
- ☐ Comment (optional)

....

....

....

D.9 How much concern do you have about the effects of extreme heat on your health?

(Tick one answer)

- ☐ No concern
- ☐ Little concern
- ☐ Moderate concern
- ☐ Major concern

D.10 Compared to previous summers, did you do anything differently this summer to help you cope with the heat?

(Tick one answer)

- ☐ **No** (now go to question D.13)
- ☐ **Yes** (now continue with question D.11)

D.11 If you answered YES to question D.10, Did you do any of the following more often this summer, than in previous summers?

(Only tick those that worked best for you)

- ☐ Used an air conditioner system
- ☐ Wore cool, light cotton clothing
- ☐ Reduced physical activities/jobs
- ☐ Stayed indoors and out of the sun
- ☐ Drank more water or juice
- ☐ Went somewhere cooler
- ☐ Ate salads or lighter meals
- ☐ Used a damp cloth/swam/showered
- ☐ Drew curtains/blinds/shutters
- ☐ Wore sunhats and sunglasses
- ☐ Slept in the coolest place at home
- ☐ **Something else?** (Please comment)

....

....

D.12 Why did you choose these different things?

____

____

D.13 Over the hot weather this summer, how often did others make contact with you or you with them?

(Tick one answer)

- ☐ Often
- ☐ Most of the time
- ☐ Sometimes
- ☐ Never

D.14 In terms of having supplies of essential items at home (E.g. food, drink and groceries, medications, household sundries), **how prepared did you feel you were for the extreme heat days this summer?**

(Tick one answer)

- ☐ Not prepared
- ☐ A little prepared
- ☐ Reasonably well prepared
- ☐ Very well prepared

E. Heat Health

E.1 **If you were not feeling well because of the heat this summer, did you contact someone from your family, friends or neighbours for help?**

(Tick one answer)

- ☐ **No** (now go to question E.3)
- ☐ **Yes** (now continue with question E.2)

E.2 **If you answered YES to question E.1, Why did you need help?** (Comment)

....

....

....

E.3 **If you were not feeling well because of the heat this summer, did you seek help from medical professionals?**

(Tick one answer)

- ☐ **No** (now go to question E.5)
- ☐ **Yes** (now continue with question E.4)

E.4 **If you answered YES to E.3, what concerned you enough to seek medical assistance**

....

....

....

E.5 **Did you experience any of the following during the hot weather this summer?**

(Tick as many as apply to you)

- ☐ **Anxiety**
- ☐ **Loss of balance / feeling dizzy**
- ☐ **A fall**
- ☐ **Headache**
- ☐ **Shortness of breath**
- ☐ **Heat stress**
- ☐ **Heart condition**
- ☐ **Renal / kidney condition**
- ☐ **Something else (*Specify*)**

....

....

E.6 **How much were you affected by the extreme hot weather this summer?**
(Tick one answer)

- ☐ **Not affected at all**
- ☐ **A little affected**
- ☐ **Somewhat affected**
- ☐ **Badly affected**

E.7 **Do you recall the SES (State Emergency Services) issuing "Extreme Heat Warnings" this summer?**

(Tick one answer)

- ☐ **No**
- ☐ **Yes**

E.8 **In your opinion, do you feel that the amount of Emergency related messages you received this summer was,**

(Tick one answer)

- ☐ **Just right**
- ☐ **Too much**
- ☐ **Not enough**
- ☐ **Other/Comment:** ....

....

....

E.9 **During this summer, which of the following sources did you use to receive heat-related information?**

(Tick as many as apply)

- ☐ **Television**
- ☐ **Radio**
- ☐ **Newspaper (printed)**
- ☐ **Newspaper (on computer)**
- ☐ **Computer Websites**
- ☐ **Facebook**
- ☐ **Printed Material(posted or picked up)**
- ☐ **Twitter**
- ☐ **Other/Comment:** ....

....

Government of South Australia
SA Health

HHM 2014

THE UNIVERSITY OF ADELAIDE AUSTRALIA

(For office use only :)
(ID.......... Type .... Date ....)

Z. Demographics

Z.1 Family Name: ______________Initial:_____

Z.2 Suburb/town: ______________Postcode:__

Z.3 What year were you born? __________

Z.4 What is your gender?

- ☐ Female
- ☐ Male

Z.5 Phone: ___________________________

Z.6 Mobile: __________________________

Z.7 This concludes the research survey. The University of Adelaide, Public Health, and SA Health thank you for taking part in this important work. Your participation will assist us to learn more about extreme heat-related problems and how we can deal with them.

Z.8 Your privacy is assured and to maintain confidentiality, questionnaire answers and your details are kept in a locked cabinet. Before the researchers analyse the completed questionnaires, all information that can identify you is removed. You will not be identifiable from any published results.

Z.9 Please indicate if you would be interested to be involved in further research and agree to have your contact details kept on file. We can then ask whether you wish to be part of future studies.

- ☐ Yes
- ☐ No
- ☐ Not sure/undecided

Z.10 If you wish to add something please feel free to write your comment below.

....
....
....
....
....
....
....
....

PLEASE USE THE

REPLY PAID ENVELOPE

TO RETURN THIS

QUESTIONNAIRE PAPER.

(THE POSTAGE IS PAID)
